# Regulation of LINE-1 Elements by miR-128 Is Not Conserved in Mouse Embryonic Stem Cells

**DOI:** 10.3389/fgene.2018.00683

**Published:** 2018-12-20

**Authors:** Maxime Bodak, Jian Yu, Constance Ciaudo

**Affiliations:** Department of Biology, Swiss Federal Institute of Technology Zurich, IMHS, Zurich, Switzerland

**Keywords:** LINE-1, miR-128, mouse embryonic stem cell, RNA interference, Dicer

## Introduction

With respectively 560,000 and 660,000 copies, Long INterspersed Element-1 (LINE-1 or L1) are the main component of the human and mouse genomes (Lander et al., 2001; Waterston et al., 2002). L1s are transposable elements encoding the proteins required for their own mobility and using an RNA intermediate for their retrotransposition (Ostertag and Kazazian, 2001). The vast majority of L1 sequences are inactive, however, some copies—around 100 in human and 3,000 in mouse—retained their ability to retrotranspose (Goodier et al., 2001; Beck et al., 2010). Due to their abundance, and their capacity to mobilize their own RNAs or other cellular RNAs (i.e., Alu elements), L1s have been shown to actively participate to the evolution of the structure and organization of the genome in which they can expand (Feschotte and Pritham, 2007; Cordaux and Batzer, 2009; Pereira et al., 2009). This L1-mediated reshape of the genomic landscape can have beneficial or detrimental outcomes, such as a positive impact on genome variability or resulting in gene disruption leading to diseases (Beck et al., 2011; Kazazian and Moran, 2017). To protect their integrities, genomes have acquired a multitude of mechanisms to regulate L1s through evolution (Bodak et al., 2014; Goodier, 2016). Concisely, mammalian L1 expression and mobility are repressed via DNA methylation in somatic cells, and via the PIWI-piRNA pathway in germ cells. However, both mechanisms are absent at the blastocyst stage, indicating potential new regulation pathways during mammalian early development (Ohnishi et al., 2010; Suh and Blelloch, 2011; Lee et al., 2014).

Since the last decade, several lines of evidence support a role for RNA interference (RNAi) pathway effector proteins in L1 regulation (Soifer et al., 2005; Yang and Kazazian, 2006; Heras et al., 2013). Using mouse Embryonic Stem Cells (mESCs), we recently showed the involvement of the RNAse III DICER in the control of L1 expression and retrotransposition (Bodak et al., 2017a). In mammals, the protein DICER is the central player of RNAi pathways and is necessary for the production of canonical and non-canonical microRNAs (miRNAs), and small interfering RNAs (siRNAs) (Kim et al., 2009; Bodak et al., 2017b). The deletion of *Dicer* in mESCs led to a strong upregulation of L1s at the RNA and protein levels, as well as a weak but significant increase of L1 mobilization rate (Bodak et al., 2017a). Our results revealed DICER and RNAi pathways as new regulators of L1 expression during early development. In line with our work, the Pedersen laboratory identified the canonical miRNA miR-128 as both, direct and indirect, L1 repressor in human cells (Hamdorf et al., 2015; Idica et al., 2017). In a first study, they identified and characterized miR-128 as a direct L1 repressor via a non-canonical binding site in the L1_ORF2 RNA, leading to the repression of full-length L1 RNAs and a decrease in retrotransposition events (Hamdorf et al., 2015). More recently, the same laboratory identified another binding site of miR-128 in the 3′UTR of *Tnpo1* mRNA, a nuclear import factor involved in the L1 mobilization process (Idica et al., 2017). They demonstrated that *Tnpo1* repression via miR-128 inhibits L1 retrotransposition by restricting L1 ribonucleoproteins nuclear import (Idica et al., 2017). Therefore, these two studies implicate the canonical microRNA pathway in the regulation of L1 elements via direct and indirect molecular mechanisms.

Both studies were conducted in human cellular systems. The structural differences between human and murine L1s (Bodak et al., 2014), and the great variability of L1 loci transcriptional activity observed between human somatic cell types (Philippe et al., 2016) limit the extension of a regulatory mechanism from one cell type to another, or between species. Consequently, it cannot be simply assumed that miR-128 is repressing L1s during mouse early development. Thus, we investigated if miR-128 also regulate murine L1s in mESCs. First, we assessed miR-128 expression in mESCs using transcriptomics and molecular approaches. Due to the low expression of miR-128 in this system, we subsequently overexpressed miR-128 using mimic microRNA and monitored L1 expression in the corresponding transfected mESCs. The upregulation of miR-128 in mESCs had no effect on L1 expression at RNA and protein levels and did not impact mouse *Tnpo1* mRNA levels. Therefore, we concluded that miR-128 is not involved in L1 repression in mESCs and that its regulatory function is not conserved between all mammalian cellular contexts.

## Material and Methods

### Culture of mESCs

E14TG2a (ATCC CRL-1821) line has been used as wild-type (WT) mESCs. Cells were cultured in DMEM (Invitrogen) supplemented with 15% of a selected batch of FBS (Gibco) tested for optimal mESC growth, 1,000 U/mL of LIF (EMD Millipore), 0.1 mM of 2-β-mercaptoethanol (Thermo Fisher Scientific), 0.05 mg/mL of streptomycin, and 50 U/mL of penicillin (Sigma-Aldrich). Cells were grown on 0.2% gelatin-coated cell culture–grade plastic dishes in the absence of feeder cells. All cells were grown at 37°C in 8% CO_2_, and the culture medium was changed daily (Jay and Ciaudo, 2013; Bodak and Ciaudo, 2016).

### Small RNA-Seq Data Analysis

Total cellular RNA was extracted from 1 × 10^6^ mESC pellets using TriZOL Reagent (Thermo Fisher Scientific). The quality of isolated RNA was determined with a Bioanalyzer 2100 (Agilent Technologies). The small RNA libraries were prepared using the Illumina TruSeq Small RNA library preparation kit according to the standardized manufacturer protocol. The sequencing was performed on an Illumina Hiseq 2500 sequencer by the Functional Genomic Center Zurich (Switzerland). Adaptors in the small RNA-seq reads were removed using Cutadapt (v1.8.1) (Martin, 2011). Reads ranged from 17 to 30 nucleotides (nt) were taken as input for mapping against mouse genome (mm10) using STAR (v2.4) (Dobin et al., 2013). No mismatches were allowed for reads shorter than 20 nt, and only 1 mismatch for reads longer than 20 nt. Aligned reads were summarized for each miRNA using featureCounts (v1.5.0) (Liao et al., 2014) with default settings, using the miRNA annotation file from miRBase V21 (Kozomara and Griffiths-Jones, 2014). Read counts were also summarized for other small RNAs, including tRNAs, small nuclear RNAs (snRNAs) and small nucleolar RNAs (snoRNAs) using featureCounts (v1.5.0) (Liao et al., 2014) and annotation file from Gencode (M9) (Harrow et al., 2006). For normalization, snRNAs, snoRNAs, and tRNAs were used as controls by function estimateSizeFactors from DESeq2 (v1.10.1) (Love et al., 2014). Complete small RNA sequencing data are available on the NCBI GEO database (GEO: GSE80454 for WT and GEO: GSE116452 for *Dicer*_KO mESCs). WT_1 sample is referred as sRNA_E14_A [miRNA-seq] (GSM2126246) and the WT_2 sample is referred as sRNA_E14_B [miRNA-seq] (GSM 2126247). The *Dicer*_KOΔ23 sample is referred as *Dicer*_KO1 (GSM3231589) and the *Dicer*_KOΔ13 sample is referred as *Dicer*_KO2 (GSM3231590). The raw count of all miRNAs in WT and *Dicer*_KO mESCs are listed in Supplementary Table 1.

### Transient Transfection of miRNA Mimic

WT mESCs were transfected into six-well plates with 60 nM of mimic miR-128 miRNA (Dharmacon^TM^ C-310398-07-002, mature sequence: 5′ UCA CAG UGA ACC GGU CUC UUU 3′) or scramble control (Dharmacon^TM^ CN-001000-01-05) using Lipofectamine^®^ RNAi max transfection reagent (Thermo Fisher Scientific) following the manufacturer's instructions (Protocol Pub. No. MAN0007825 Rev. 1.0). 200,000 WT mESCs per well were seeded 24 h before transfection. Medium was changed 24 h after transfection. Cells were harvested 48h after transfection.

### RT-qPCR Analysis

Total cellular RNA was extracted from 1 × 10^6^ mESC pellets using TriZOL Reagent (Thermo Fisher Scientific). Extract quality was verified by loading 1 μg total RNA on a 1% agarose gel (Bodak and Ciaudo, 2016).

For mRNA quantification, a total of 1 μg total RNA was treated with DNase (RQ1 Rnase-Free DNase kit; Promega) and then reverse transcribed according to the manufacturer's protocol using a GoScript Reverse transcription kit (Promega). After the reverse transcription reactions, cDNA products were diluted in distilled water (1:5). For each extract, PCR on the *Rrm2* gene was performed, before and after reverse transcription treatment, to ensure the absence of genomic DNA contamination. Quantification of expression levels was performed on a Light Cycler 480 (Roche) using 2 μL of the diluted cDNAs and the KAPA SYBR FAST qPCR kit Optimized for Light Cycler 480 (KAPA Biosystems) (Bodak and Ciaudo, 2016).

For miRNA quantification, 1 μg total RNA was reverse transcribed using the miScript II Reverse Transcription kit (Qiagen) according to the manufacturer's instructions. After the reverse transcription reactions, cDNA products were diluted in distilled water (1:5). Quantification of expression levels was performed on a Light Cycler 480 (Roche) using 2 μL of the diluted products, the KAPA SYBR FAST qPCR kit Optimized for Light Cycler 480 (KAPA Biosystems) and miScript Universal Primer (Qiagen). Differences between samples and controls were calculated based on the 2^−Δ*CT*^ method. Quantitative RT-PCR assays were performed in triplicate (Jay and Ciaudo, 2013; Bodak and Ciaudo, 2016). All the primers used for the RT-qPCR assays are listed in Supplementary Table 2.

### Low Molecular Weight Northern Analysis

Total cellular RNA was extracted from 1 × 10^6^ mESC pellets using TriZOL Reagent (Thermo Fisher Scientific). Extract quality was verified by loading 1 μg total RNA on a 1% agarose gel. A total of 10 μg total RNA was resuspended in 30 μL final of 50% deionized formamide, loaded on a 17.5% acrylamide gel (30% acrylamide/bis solution 19:1; Bio-Rad Laboratories), ran 3 h at 100V, blotted for 1 h on a nylon membrane (Amersham Hybond-NX; GE Healthcare) in 0.5 × TBE (Tris/borate/EDTA) buffer at 25 V and 1.5 mA per square centimeter of membrane in a semidry system. Membranes were then UV cross-linked (1,200 mJ/cm^2^ optimal cross-linking setting in a Spectrolinker XL-1500 UV crosslinker). Prehybridizations and hybridizations were both performed in PerfectHyb Plus Hybridization Buffer (Sigma-Aldrich) at 42°C. All washes were performed in SSC 2 × , SDS 0.1%. Radioactive signals were detected with an FLA-7000 device (Fujifilm). For subsequent reprobing, membranes were stripped with boiling 0.1% SDS. miRNA and U6 probes were generated by labeling specific oligonucleotides at the 5′ end using T4 polynucleotide kinase (New England Biolabs, Inc.) and 25 μCi γ[^32^P]-ATP (3,000 Ci/mmol) and following the manufacturer's instructions. Probes were then purified on Illustra MicroSpin G-25 Columns (GE Healthcare) (Jay and Ciaudo, 2013). All of the probes used for miRNA Northern blots are listed in Supplementary Table 2.

### Immunoblotting Analysis and Antibodies

Total cellular protein was extracted from 1 × 10^6^ mESC pellets using a NP-40–based lysis buffer (1% NP-40, 137 mM NaCl, 20 mM Tris-HCl, and 1 mM EDTA) complemented with EDTA-free protease inhibitor cocktail (Roche). Protein concentrations were determined by Bradford Assay (Bio-Rad Laboratories). For each sample, 10 μg total cellular protein was separated in 10% SDS-PAGE gels and transferred on polyvinylidene fluoride membranes. All the antibodies used for the immunoblot assays are described in Supplementary Table 3. Immunoblots were developed using the Clarify Western ECL substrate (Bio-Rad Laboratories) kit and detected using an imaging system (ChemiDoc MP; Bio-Rad Laboratories) (Bodak and Ciaudo, 2016). All membranes were stained with Coomassie Blue to ensure equal loading.

## Results

### miR-128 Is Lowly Expressed in mESCs

**Figure 1:**
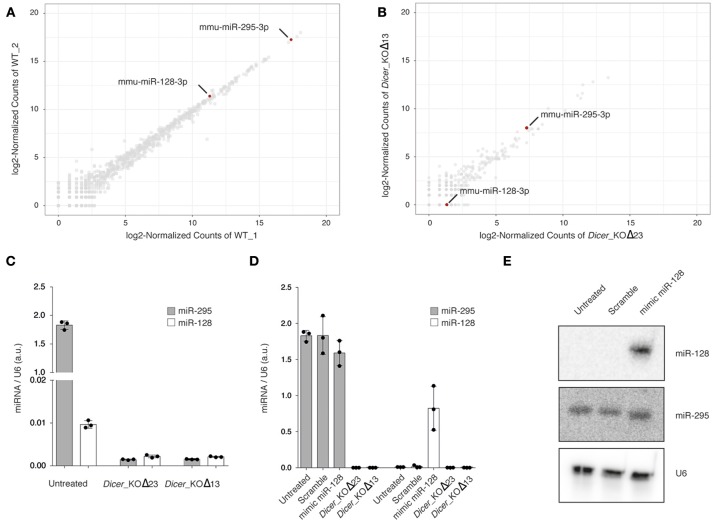
**(A,B)** Scatterplot of normalized miRNA abundance in wild type (WT) and *Dicer*_KO mESCs, respectively. mmu-miR-128-3p and mmu-miR-295-3p are highlighted in red. **(C)** miRNA RT- qPCR analysis of miR-295 and miR-128 expression in WT and *Dicer*_KO mESCs, **(D)** miRNA RT- qPCR analysis of miR-295 and miR-128 expression in WT, 48 h after transfection of mimic miR-128 or scramble mimic miRNA. The data are shown as arbitrary unit (a.u.) after normalization to U6. Data are represented as mean ± SD (*n* = 3). **(E)** Northern blot analysis using WT mESCs, 48 h after transfection of mimic miR-128 or scramble mimic miRNA, total RNA extract probed with specific miR-295 and miR-128 probes. Samples were probed with a U6-specific probe as loading control. Representative blot of three independent experiments is shown. Original blots are available in Supplementary Figure 1.

To assess global miRNA expression in WT mESCs, we carried out small RNA sequencing with Illumina HiSeq 2500 (Figure 1A and Supplementary Table 1). The data analysis revealed a weak expression of miR-128 compared to miR-295, a stem cell specific miRNA, known to be well-expressed in mESCs (Houbaviy et al., 2003; Ciaudo et al., 2009). As a control experiment, we also performed small RNA sequencing from *Dicer*_KO mESCs(Bodak et al., 2017a), unable to produce miRNAs, and observed, as expected, a strong downregulation of all microRNAs, including miR-295 and miR-128 (Figure 1B and Supplementary Table 1). In order to validate these observations, we performed miRNA RT-qPCR using specific primers for miR-295 and miR-128 (Figure 1C) and confirmed the low expression of miR-128 compared to miR-295 in WT mESCs. Both miRNA expression were strongly reduced in *Dicer_*KO mESCs (Figure 1C).

To circumvent the low expression of miR-128 in mESCs, we transfected WT mESCs with mimic miR-128 or with a scramble mimic miRNA. We harvested the cells 48 h after transfection and assessed the expression of both miRNAs. The enrichment for miR-128 in transfected cells was detectable by RT-qPCR and Northern Blot using specific primers and probes, and had no impact on the level of miR-295 (Figures 1D,E).

Taken together these experiments indicate that miR-128 is very low expressed in WT mESCs, and that it is possible to artificially increase its amount using a mimic miRNA approach.

### Transient Transfection of Mimic miR-128 in WT mESCs Does Not Impact L1 Expression

**Figure 2:**
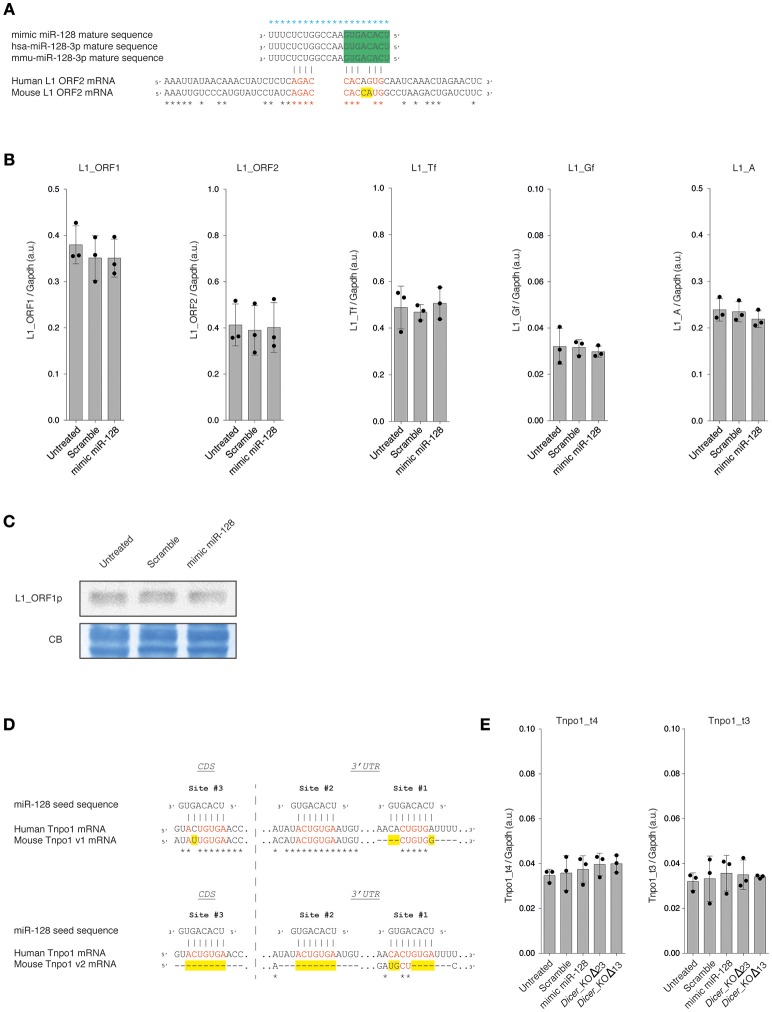
**(A)** Alignment of human and murine miR-128 mature sequences and miR-128 binding sites in human (GenBank: AH005269.2) and murine (L1Base: ID1048) L1_ORF2 mRNA. Alignments were performed using T-Coffee (Notredame et al., 2000). Blue stars indicate identity between the miR-128 mature sequences. Black stars indicate identity between the human and mouse L1_ORF2 mRNA consensus sequences. Identical nucleotides between human and mouse miR-128 seed sequences in L1_ORF2 are indicated in red and mismatches in yellow. The seed sequence of miR-128 is indicated in green. **(B)** RT- qPCR analysis of overall L1s (L1_ORF1 and L1_ORF2) and specific L1 subfamily (L1_Tf, L1_Gf, and L1_A) mRNAs in WT mESCs, 48 h after transfection of mimic miR-128 or scramble siRNAs. The data are shown as arbitrary unit (a.u.) after normalization to the *Gapdh* housekeeping gene. Data are represented as mean ± SD (*n* = 3). **(C)** Immunoblot analysis of L1_ORF1 protein levels in WT mESCs, 48 h after transfection of mimic miR-128 or scramble siRNAs. Membranes were stained with Coomassie Blue (CB) to ensure equal loading. Representative blot of three independent experiments is shown. Original blots are available in Supplementary Figure 2. **(D)** Alignment of miR-128 seed sequences and miR-128 binding sites in human (ENST00000337273.9) and murine (v1: uc007rpf.1 and v2: uc007rph.1) *Tnpo1* mRNA sequences. Alignments were performed using T-Coffee (Notredame et al., 2000). Black stars indicate identity between the *Tnpo1* mRNA sequences. Identical nucleotides between human and mouse miR-128 binding sites are indicated in red and mismatches in yellow. CDS: coding sequence. 3′UTR: 3′ untranslated region. **(E)** RT- qPCR analysis of *Tnpo1* mRNAs in WT mESCs, 48 h after transfection of mimic miR-128 or scramble siRNAs. The data are shown as arbitrary unit (a.u.) after normalization to the *Gapdh* housekeeping gene. Data are represented as mean ± SD (*n* = 3).

The mature sequence of the canonical miR-128-3p is perfectly conserved between mouse and human species (miRbase release 22 accession number MIMAT0000140 and MIMAT0000424, respectively) (Kozomara and Griffiths-Jones, 2014). This microRNA was first described to repress L1 expression through a non-canonical seed binding site located in the coding region (ORF2) of the human L1 mRNA (Hamdorf et al., 2015). Using the same references of consensus sequences for L1_ORF2 (mouse L1Base: ID1048 and human GenBank: AH005269.2) from Hamdorf and colleagues, we established that mouse and human L1_ORF2 share 68.95% of identity. Moreover, the mouse L1_ORF2 contains an additional mismatch in the putative non-canonical seed sequence interaction with miR-128 (Figure 2A). However, considering the high number of potential active L1 copies in the mouse genome (Goodier et al., 2001; Beck et al., 2010) and that a consensus sequence has been used for this alignment, we could not exclude that a subset of these copies features a similar non-canonical or canonical miR-128 seed binding site.”

Using a similar mimic miRNA approach as described previously (Hamdorf et al., 2015; Idica et al., 2017; Huang et al., 2018), we investigated if the overexpression of miR-128 might impact L1 expression in mESCs. WT mESCs were transfected with mimic miR-128 or with a scramble miRNA and harvested after 48 h. We first assessed L1 mRNA expression by RT-qPCR. Using primers common to all L1s (L1_ORF1 and L1_ORF2), we did not detect any differences in the expression of L1 RNA levels between WT mESC non-transfected, transfected with the miR-128 mimic or with a scramble mimic miRNA (Figure 2B). Similar observations were made when using primers specific for each murine active L1 sub-families (L1_Tf, L1_Gf and L1_A) (Figure 2B) (Bodak and Ciaudo, 2016).

Then, we monitored L1 protein level by immunoblot analysis using an antibody specific for the ORF1 protein of murine L1s. As shown in Figure 2C, we did not notice any variation in ORF1 protein level among the different samples. Taken together these results show that miR-128 transient overexpression does not impact L1 RNA and protein expression in mESCs.

### Transient Transfection of Mimic miR-128 in WT mESCs Does Not Impact *Tnpo1* mRNA Levels

In a more recent study, the Pedersen laboratory revealed that miR-128 also controls L1 retrotransposition by directly regulating the level of nuclear import factor Transportin-1 (TNPO1) (Idica et al., 2017). The TNPO1 (also named Karyopheryn-ß2 or Importin-ß2) belongs to the ß-Karyopheryn family, a class of protein involved in active nucleo-cytoplasmic transport by binding to diverse nuclear localization signal sequences (Twyffels et al., 2014). The TNPO1 has been shown to participate to the nuclear import of viral, ribosomal, and histone proteins (Twyffels et al., 2014).

In human, three *Tnpo1* transcript variants can be distinguished (UCSC Genome Browser on Human Dec. 2013 GRCh38/hg38 Assembly: ENST00000337273.9, ENST00000506351.6 and ENST00000523768.5). Among them, only the transcript variant ENST00000337273.9 features three miR-128 binding sites characterized in Idica et al. (2017): one in the coding sequence (Site #3) and two in the 3'UTR (Site #1 and #2) (Figure 2D). The two other variants feature only one miR-128 binding site (Site #3). In mouse, five *Tnpo1* transcript variants are described (UCSC Genome Browser on Mouse Dec.2011 GRCm38/mm10 Assembly). For the variants uc007rpd.1, uc007rpe.1, and uc007rpf.1, the miR-128 binding sites #2 and #3 appeared to be well conserved between human and mouse with only one additional mismatch in the mouse canonical seed binding sequence (Figure 2D), however the Site #1 is poorly conserved (Figure 2D). The variant uc007rpg.1 contains only the Site #3 (one mismatch). Interestingly, the transcript variant uc007rph.1 does not have any miR-128 canonical binding sites, as sites #2 and #3 are absent of the 3′UTR and site #1 is very poorly conserved (Figure 2D).

In order to determine if miR-128 regulates *Tnpo1* in mESCs, we assessed *Tnpo1* mRNA levels by RT-qPCR using two distinct sets of primers: t4 (uc007rpd.1, uc007rpe.1, uc007rpf.1 and uc007rph.1) and t3 (uc007rpd.1, uc007rpe.1, uc007rpf.1 and uc007rpg.1) (Figure 2E). We did not detect any differences in expression of *Tnpo1* RNA levels between WT mESC non-transfected, transfected with the miR-128 mimic or with a scramble mimic miRNA (Figure 2E). Taken together these results show that miR-128 transient overexpression does not impact *Tnpo1* mRNA levels in mESCs.

## Discussion

In this report, we showed using distinct approaches (small RNA sequencing, miRNA RT-qPCR and Northern Blot) that miR-128 is expressed at low level in WT mESCs. We subsequently revealed that the non-canonical miR-128 seed sequence on murine L1_ORF2 RNA contains an additional mismatch compared to human L1_ORF2 sequence. This one base pair difference might explain that the transient overexpression of miR-128 had no effect on L1 RNA and L1 protein levels in WT mESCs. Considering its low expression, the peculiarity of its target site and lack of effect of its overexpression, we conclude that miR-128 has potentially no direct regulatory function on L1 expression in mESCs. However, even if L1 RNA is bicistronic in both species, only murine L1 RNA has been shown to have an internal ribosome entry site upstream to each ORF (Li et al., 2006). Therefore, in order to definitely rule out the direct regulation of L1 by miR-128 in mESCs, the level of L1_ORF2 protein, after the transient overexpression of miR-128, would need to be assessed. Unfortunately, we were not able to detect L1_ORF2 protein levels due to the lack of a specific mouse antibody.

Additionally, we investigated the possible down-regulation by miR-128 of the nuclear import factor TNPO1. We unveiled that in mouse, no *Tnpo1* transcript variants feature the preferential miR-128 biding site (Site #1) located in the 3′UTR, described in Idica et al. (2017). Finally, we demonstrated that miR-128 transient overexpression had no impact on *Tnpo1* mRNA levels. Therefore, it emerged that *Tnpo1* mRNA levels are not regulated by miR-128 in mESCs. Discrepancies between miRNA target regulation in specific cell types and mESCs are not unusual, as the aforementioned appeared to have shorter 3′UTR (Ji et al., 2009).

Taken together, we showed that miR-128 overexpression did not alter L1 RNA, L1 protein and *Tnpo1* RNA levels in mESCs, and that its dual L1 regulatory mechanism is not conserved between human cells and mESCs.

## Data Availability

The complete small RNA sequencing data sets are available on the NCBI GEO database (GEO: GSE80454 for WT and GEO: GSE116452 for *Dicer*_KO mESCs). WT_1 sample is referred as sRNA_E14_A [miRNA-seq] (GSM2126246) and the WT_2 sample is referred as sRNA_E14_B [miRNA-seq] (GSM 2126247). The *Dicer*_KOΔ23 sample is referred as *Dicer*_KO1 (GSM3231589) and the *Dicer*_KOΔ13 sample is referred as *Dicer*_KO2 (GSM3231590).

## Author Contributions

MB and CC conceived study, performed experiments, analyzed data, and wrote the manuscript. JY contributed to bioinformatics analysis.

### Conflict of Interest Statement

The authors declare that the research was conducted in the absence of any commercial or financial relationships that could be construed as a potential conflict of interest.
