# Supplementary figures and images for: Regulation of LINE-1 Elements by miR-128 Is Not Conserved in Mouse Embryonic Stem Cells
Source: Front Genet. 2018 Dec 20;9:683. doi: 10.3389/fgene.2018.00683 (PMC6306448; doi:10.3389/fgene.2018.00683)

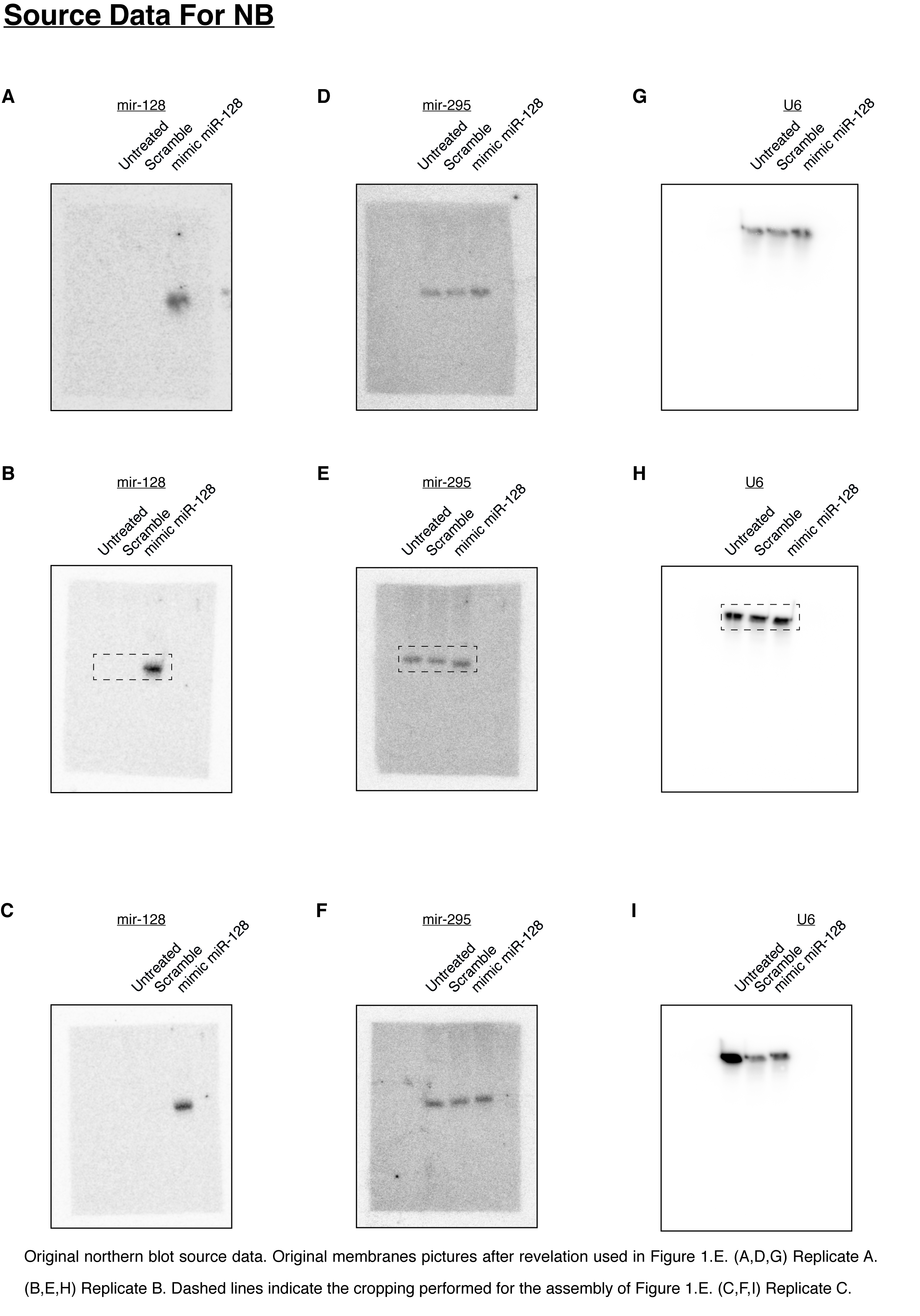

Supplement: Supplementary file 2 [file Image_1.TIF]

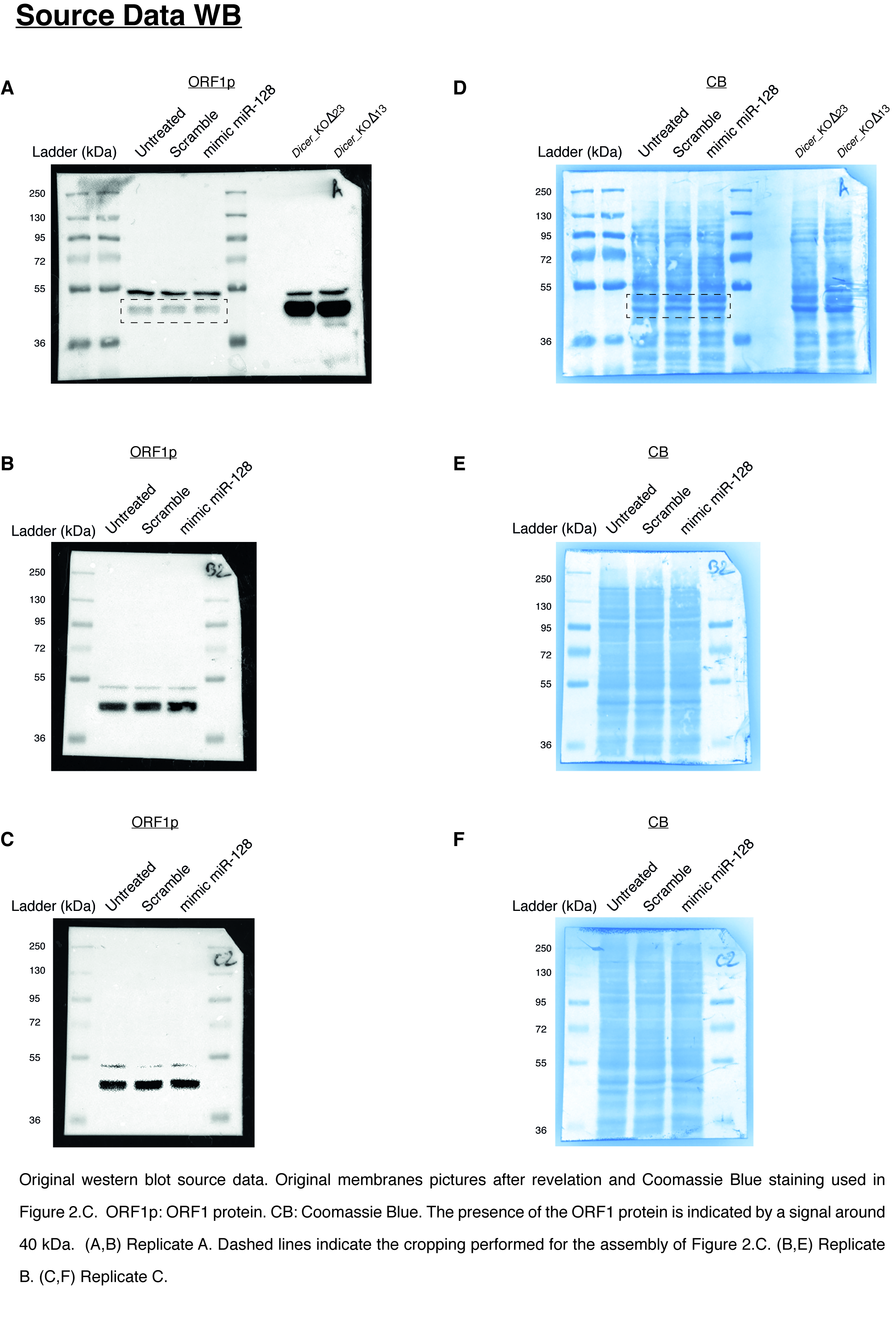

Supplement: Supplementary file 3 [file Image_2.TIF]
